# Open-path measurement of stable water isotopologues using mid-infrared dual-comb spectroscopy

**DOI:** 10.5194/amt-16-4053-2023

**Published:** 2023

**Authors:** Daniel I. Herman, Griffin Mead, Fabrizio R. Giorgetta, Esther Baumann, Nathan A. Malarich, Brian R. Washburn, Nathan R. Newbury, Ian Coddington, Kevin C. Cossel

**Affiliations:** 1 Spectrum Technology and Research Division, National Institute of Standards and Technology, Boulder, Colorado 80305, United States of America; 2 Department of Physics, University of Colorado Boulder, Boulder, Colorado 80309, United States of America

## Abstract

We present an open-path mid-infrared dual-comb spectroscopy (DCS) system capable of precise measurement of the stable water isotopologues H_2_^16^O and HD^16^O. This system ran in a remote configuration at a rural test site for 3.75 months with 60% uptime and achieved a precision of < 2‰ on the normalized ratio of H_2_^16^O and HD^16^O (δD) in 1000s. Here, we compare the δD values from the DCS system to those from the National Ecological Observatory Network (NEON) isotopologue point sensor network. Over the multi-month campaign, the mean difference between the DCS δD values and the NEON δD values from a similar ecosystem is < 2‰ with a standard deviation of 18‰, which demonstrates the inherent accuracy of DCS measurements over a variety of atmospheric conditions. We observe time-varying diurnal profiles and seasonal trends that are mostly correlated between the sites on daily timescales. This observation motivates the development of denser ecological monitoring networks aimed at understanding regional- and synoptic-scale water transport. Precise and accurate open-path measurements using DCS provide new capabilities for such networks.

## 1 Introduction

A better understanding of water transport on different scales is necessary to understand the impacts of climate change on global water use (Jury and Vaux, 2005). A continuous record of stable water isotopologues in atmospheric water vapor and precipitation can provide a benchmark for models of water transport ranging from global circulation to field-scale mass balance (Galewsky et al., 2016; Al-Oqaili et al., 2020; Welp et al., 2008; Good et al., 2015; Dansgaard, 1964; Craig, 1961). For many types of water isotopologue measurements, it is necessary to have a network of accurate sensors. Here, we demonstrate accurate, long-term open-path dual-comb spectroscopic measurements of H_2_^16^O ($H_{2}O$) and the deuterium-substituted isotopologue HD^16^O (HDO) in atmospheric water vapor. The ratio of HDO to $H_{2}O$, referred to as δD, is characterized using the standard definition (Galewsky et al., 2016):

(1) $$\delta D=\frac{R_{\text{measured}}}{R_{VSMOW}}-1,$$

where $R_{\text{measured}}=[HDO]/\left[H_{2}O\right]$ is the measured isotopologue ratio (ratio of absolute concentrations) and $R_{VSMOW}=0.0003115$ is the standard isotopologue ratio according to the Vienna Standard Mean Ocean Water (VSMOW) scale. Normally, δD is expressed as a “per mil” number (‰). Values of δD in nature fall in the range from −500‰ to 0‰ with spatial and temporal variation driven by mass fractionation during condensation and evaporation processes. By monitoring δD, it is possible to simultaneously track local evapotranspiration in the planetary boundary layer and synoptic-scale water transport (Noone et al., 2013; Galewsky et al., 2016).

Currently, most measurements of stable water isotopologues in atmospheric vapor rely on point sensor networks (Finkenbiner et al., 2022; Fiorella et al., 2021; Wei et al., 2019; Aemisegger et al., 2012) that can be difficult to calibrate (Bailey et al., 2015; Rambo et al., 2011) and utilize relatively expensive technology. Cryogenic discrete point sampling and cavity-enhanced optical absorption point sensors achieve accuracy on the order of 2‰ or less (Galewsky et al., 2016) but require careful, frequent calibration. Long open-path measurements could enhance the capabilities of sensor networks if the measurements have high precision and accuracy. In general, open-path sensing techniques for atmospheric gases avoid sampling biases inherent in extraction systems (inlets, tubing, pumps, filters, etc.), which is especially advantageous for “sticky” gases such as water vapor or ammonia. In addition, the spatial averaging provided by open-path techniques enables clearer comparisons between field measurements and atmospheric models (typically computed on kilometer-scale grids) (Griffith et al., 2018). Open-path Fourier transform infrared (FTIR) spectroscopy for δD retrieval has been reported previously (Wang et al., 2012; Griffith et al., 2018), but sources of error can arise in open-path FTIR related to the instrument line shape and limited spectral resolution of deployable systems (Lin et al., 2020), and have not been investigated for δD.

The use of a open-path dual-comb spectroscopy (DCS) (Rieker et al., 2014; Waxman et al., 2017; Giorgetta et al., 2021; Cossel et al., 2021) is promising for stable water isotopologue measurements due to the combination of broad spectral bandwidth, high spectral resolution, high brightness, and spatial coherence, which enables kilometer-scale measurements of multiple trace species simultaneously (Coddington et al., 2016). Open-path DCS is an emerging Fourier spectroscopy technique based on sampling the atmosphere with laser frequency combs. This technique achieves high spectral resolution (here, the spectral point spacing is 200MHz or 0.0067cm^−1^) with no instrument line shape while also spanning hundreds of wavenumbers (Waxman et al., 2017). Several demonstrations have already shown the potential of laboratory DCS for isotopologue measurements (Muraviev et al., 2018; Vodopyanov, 2020; Parriaux et al., 2022). Open-path DCS optical paths can be easily reconfigured using remotely controlled telescopes to adjust to measurement conditions (Coburn et al., 2018) and can be spatially scanned using mobile reflectors (Cossel et al., 2017), which could enable better tomographic characterization of evaporation and transpiration in agricultural systems and natural ecosystems (Welp et al., 2008).

In this work, we demonstrate a mid-infrared (MIR) open-path DCS system capable of δD measurement with a precision of 1.2‰ with 3000 s of averaging. This precision is sufficient to capture diurnal changes in δD at our test measurement site (Platteville Atmospheric Observatory; PAO), which can range from 10‰ to 100‰. We demonstrate measurements over a 3.75-month-long measurement period with 60% uptime from fall into winter through a variety of meteorological conditions. We compare these DCS δD measurements to data from two nearby National Ecological Observatory Network (NEON) sites: the Central Plains Experimental Range (CPER) and Niwot Ridge (NIWO) (Finkenbiner et al., 2022). The difference in δD values measured by DCS at PAO and by the point sensor at CPER has a mean of less than 2‰ and a standard deviation of 18‰. The NIWO site is in an alpine ecosystem, whereas PAO is located in a great plains ecosystem, so one would not expect a similar level of agreement. A comparison between δD at PAO and NIWO demonstrates that gradients of δD with altitude near mountainous regions are strong but not omnipresent. Finally, we look at some features of the data series including diurnal profiles and Keeling curves for a few time periods to demonstrate subtle differences between PAO and CPER meteorology. These results demonstrate that MIR open-path DCS can be a viable tool for water isotopologue measurements and can provide data that complement and extend current monitoring capabilities.

## 2 Experimental setup

### 2.1 MIR DCS generation

**Figure 1.**
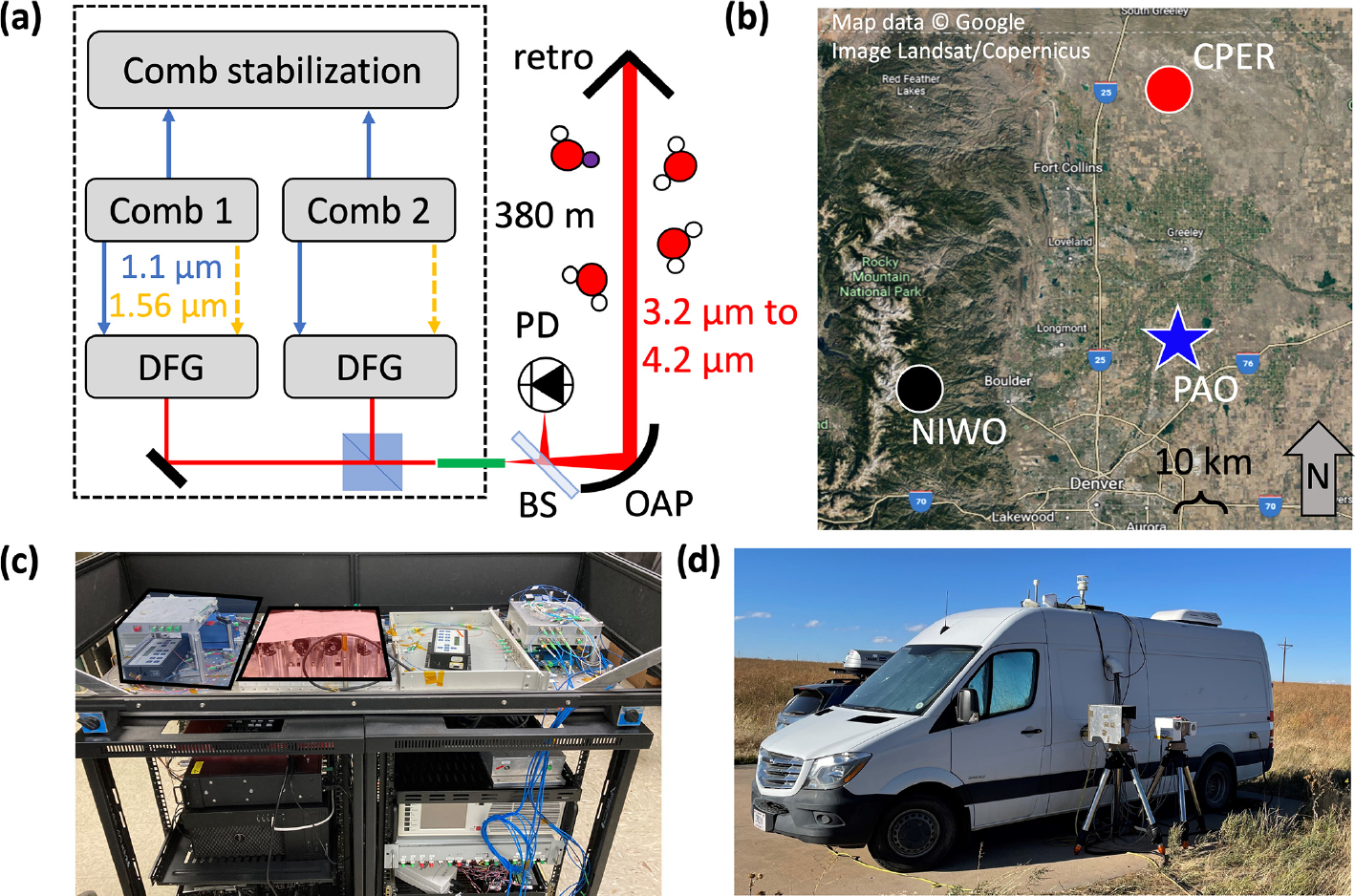
MIR DCS experimental setup. (**a**) Schematic of MIR DCS system with dashed line indicating the boundary of the mobile DCS van. DFG: difference frequency generation. PD: photodetector. BS: beam splitter. OAP: off-axis parabolic mirror. Red lines indicate free-space MIR beam paths. Blue lines indicate supercontinuum light near 1150 nm. Dashed orange lines indicate light near 1560 nm. Green line indicates InF_3_ single-mode fiber. (**b**) Satellite image of northern Colorado Front Range Urban Corridor with PAO (blue star), CPER (red circle), and NIWO (black circle) sites marked. The NIWO and CPER sites are both ~75 km away from the PAO site. Map data: Google. (**c**) Image of the field-deployable MIR DCS system. Optical platform containing combs (Comb 1 shaded in blue box) and DFG optics (shaded in red box) is placed on top of two electronics racks containing comb control and acquisition systems. (**d**) Image of mobile DCS van at PAO site with MIR telescope system in the foreground. (The second telescope supports parallel measurements from a near-infrared DCS system that are not discussed here.)

The deployable MIR DCS system illustrated in Fig. 1a follows the design from Ycas et al. (2020) to generate laser light spanning 3.2 to 4.2 μm while leveraging a robust fiber laser architecture. We briefly review the design below along with minor changes that enabled improved field operation of the MIR DCS system. The MIR light is produced using difference frequency generation (DFG) in aperiodically poled lithium niobate (aPPLN) waveguides seeded by near-infrared fiber frequency combs (Erny et al., 2007; Maser et al., 2017; Ycas et al., 2018). The system is based on two fully stabilized near-infrared combs (Sinclair et al., 2015) centered at 1560 nm with repetition frequencies of ~200MHz and a repetition frequency difference of 208Hz. By supplementing standard DCS locking schemes (Truong et al., 2016) with digital phase correction (Roy et al., 2012; Ycas et al., 2018), the MIR DCS system achieves high mutual coherence in field environments.

To generate the MIR light through DFG, the output of each comb is first split into two branches, as discussed in Ycas et al. (2020). One branch uses spectral broadening in highly nonlinear fiber (HNLF) to generate pulsed light spanning 1.05 to 1.2 μm, which, unlike the system reported in Ycas et al. (2020), is directly coupled into free space from the HNLF using an off-axis parabolic (OAP) mirror for improved efficiency. In the second branch, pulses are amplified at 1565 nm (~20 nm bandwidth) and temporally stretched in 5m of polarization-maintaining fiber to a pulse width of about 1 ps. The ~1.1 and 1.565 μm light from both branches is then combined on a long-pass dichroic mirror with a cutoff of 1180 nm and coupled into the aPPLN waveguide by an f=25mm anti-reflection (AR)-coated achromatic lens. The aPPLN waveguide is chirped to support broadband frequency conversion of the near-infrared light to the 3.0-to-4.0 μm region (Suchowski et al., 2009). Slow thermal or other effects that cause pulse walk-off between the two branches will reduce the MIR light generation in the aPPLN waveguide. Placing the HNLF in the first branch in an insulated metal housing and stretching the second branch pulse duration to 1 ps both greatly reduced pulse walk-off. Any residual pulse walk-off is compensated for by a fiber-coupled computer-controlled optical delay line placed in the 1.565 μm branch, which is adjusted to optimize the MIR DCS output power.

The MIR output of the aPPLN waveguide from one comb is collimated, filtered using an AR-coated germanium wedge, combined with the MIR output of the other comb on a broadband AR-coated CaF_2_ beam splitter, and coupled into InF_3_ fiber for transport to the telescope (see Fig. 1a). The spectrum coupled into fiber covers a continuous region from 3.2 to 4.2 μm, up to the CO_2_ absorption band. More than 1 mW of fiber-coupled power is available in the 3.5-to-4.0 μm region where there is a strong HD^16^O absorption band centered at 3.6 μm. A waveguide redesign or modification of the DFG approach will enable coverage from 3.0 to 5.0 μm simultaneously (Zhou et al., 2020). Such bandwidth would give access to cleaner H_2_^18^O and H_2_^16^O absorption bands in addition to the strong HDO and H_2_^16^O bands covered with the current spectral bandwidth.

### 2.2 Field deployment of the MIR DCS system

The entire MIR DCS optical system (free-space and fiber optics) is placed on a 1.2 m by 0.5 m platform, which is then stacked on two electrical rack units (each measuring 0.5 m by 0.8 m by 0.84 m) containing the pump diodes, temperature controllers, detectors, locking electronics, DCS acquisition system, computers, and power supplies (Fig. 1c). Many of these components could be further reduced in size to make the system more compact. To limit sensitivity to environmental perturbations, the actual free-space optics for dual-comb DFG only occupy a 0.35 m by 0.35 m custom breadboard. The platform and breadboard are covered by a laser eye-safe box, and the entire double rack unit (with a volume of 0.75 m^3^) is installed in the back of a van, which is then driven to the PAO site near Platteville, Colorado (40.181782° N, 104.725054° W), as shown in Fig. 1b. This rural site is in the Denver–Julesburg Basin, which is a major oil and gas production area.

**Figure 2.**
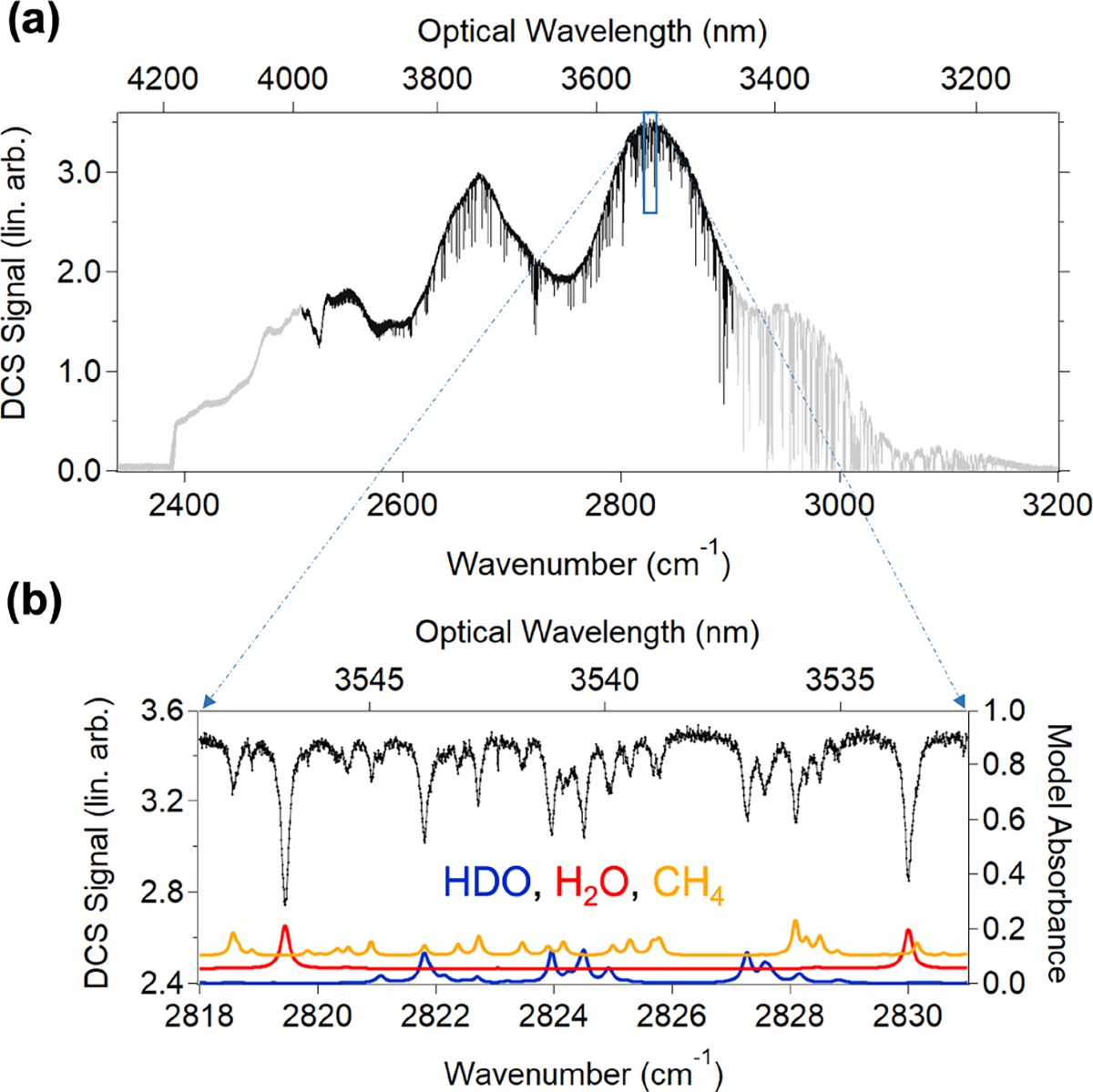
(**a**) MIR DCS spectrum (2 min average, linear arbitrary units) taken over the 760 m round trip path at PAO (grey trace). Black trace indicates region selected for spectral analysis in this work. (**b**) Zoomed-in MIR DCS spectrum from 2818 to 2831 cm^−1^. Black dots indicate DCS signal (left axis). The red and blue traces are the $H_{2}O$ and HDO absorption models, respectively, from HITRAN2020 (right axis with $H_{2}O$ offset by 0.05). The orange trace is the CH_4_ absorption model from HITRAN2020 (right axis and offset by 0.1). Although absorption from other molecules contributes additional clutter in this region, CH_4_ is the major source.

The fiber-coupled MIR DCS light is sent to a telescope located outside of the van (Fig. 1d). In the telescope (Fig. 1a), the diverging beam from the InF_3_ fiber is collimated using an f=178.55mm OAP, which results in a 6.8 cm diameter beam that can be projected over long paths. The telescope is placed on a tripod-mounted azimuth–elevation gimbal and is aimed at a 12.7 cm diameter retroreflector located 380m away. The DCS light reflected from the retroreflector returns to the telescope and is then reflected by a broadband AR-coated CaF_2_ beam splitter placed between the fiber end and the collimating OAP onto a thermoelectrically cooled HgCdTe detector. A co-axial cable transmits the detected dual-comb interferogram voltage signal back to the van where it is digitized, phase-corrected, and coherently co-added to generate one DCS spectrum every 2 min (Fig. 2a). Each of these averaged spectra is derived from ~25 000 raw unaveraged interferograms. In addition to the DCS spectrum, several other datasets are available for analysis. Meteorological data are provided by a sensor at the PAO site operated by the National Oceanic and Atmospheric Administration (NOAA, site code: PVL). These data include wind direction and speed, solar radiation, relative humidity, temperature, and precipitation. Water mixing ratios are also recorded at the site using two different commercial cavity ring-down spectroscopy (CRDS) instruments. We note that the MIR DCS system described here is also capable of precise detection of multiple species in addition to water isotopologues including primary greenhouse gases (CO_2_, CH_4_, N_2_O), air pollutants (e.g., CO), and volatile organic compounds (e.g., HCHO and C_2_H_6_).

## 3 Data analysis

### 3.1 Broadband cepstral-domain fitting of MIR DCS data

The deployable open-path MIR DCS system generates broadband, high-resolution atmospheric spectra shown in Fig. 2a that cover rovibrational absorption features from many different atmospheric species. In order to isolate water isotopologue absorption from these congested broadband spectra, one could select several “micro-windows” where traditional frequency-domain fitting techniques can successfully extract concentrations in a reliable manner (Griffith et al., 2003; García et al., 2022). In this work, we instead choose to utilize a broadband fit using cepstral-domain analysis of molecular free-induction decay (Cole et al., 2019). The cepstral transform starts with an intensity spectrum in the frequency domain which is then converted to an absorbance spectrum by taking the logarithm. This absorbance spectrum is then Fourier-transformed to yield a “cepstrum” which represents the modified molecular free-induction decay (FID) in the time (“cepstral”) domain. The cepstral-domain technique simplifies the fitting of broadband DCS data by separating the baseline structure, etalon effects, and FID into different cepstral regions. The data are fit in the cepstral domain using a 10-to-350 ps bandpass filter. The 10 ps cut-on is applied to the DCS spectra to remove baseline effects, and the 350 ps cutoff avoids etalons due to the aPPLN waveguides themselves. No other significant etalons occur between these two end points. The effect of the cepstral filter choice on the fit retrievals is discussed in Sect. 3.3. To avoid complications of fitting deep absorption features that approach the spectrum noise floor, the fit region is restricted to the weaker absorption section of the DCS spectrum ranging from 3.5 to 4.0 μm (see Fig. 2a). This region includes most of the *ν*_1_ absorption band of HDO as well as the long-wavelength end of the 2*ν*_2_ band of $H_{2}O$. The large number and strength of HDO transitions within this fit region ensure a very precise measurement of this secondary isotopologue. We note that the peak absorption strengths of HDO and $H_{2}O$ in this region are similar, a desirable trait for any optical isotopologue detection technique. Importantly, the 200MHz point spacing of the DCS spectra fully resolves the absorption lines of both HDO and $H_{2}O$ as well as cluttering species like CH_4_ (see Fig. 2b).

We model the FID signals using absorption cross-sections generated using line-by-line data from HITRAN2020 (Gordon et al., 2022). We include H_2_^16^O, HD^16^O, and H_2_^18^O absorption, but the retrieved H_2_^18^O concentration does not have sufficient precision to be useful due to the weak absorption in the fit spectral region. In addition to water, the fit model includes CH_4_, C_2_H_6_, N_2_O, CO_2_, HCHO, and HCl. Concentrations of these species can be inferred from the DCS spectra with useful precisions for analysis of ambient air. In the cepstral fit, temperature is allowed to vary while the pressure is fixed to a measured value at PAO. These parameters are used to determine the atmospheric number density according to the ideal gas law (although the isotopologue ratio $R_{\text{measured}}$ and δD are independent of the total air mass).

**Figure 3.**
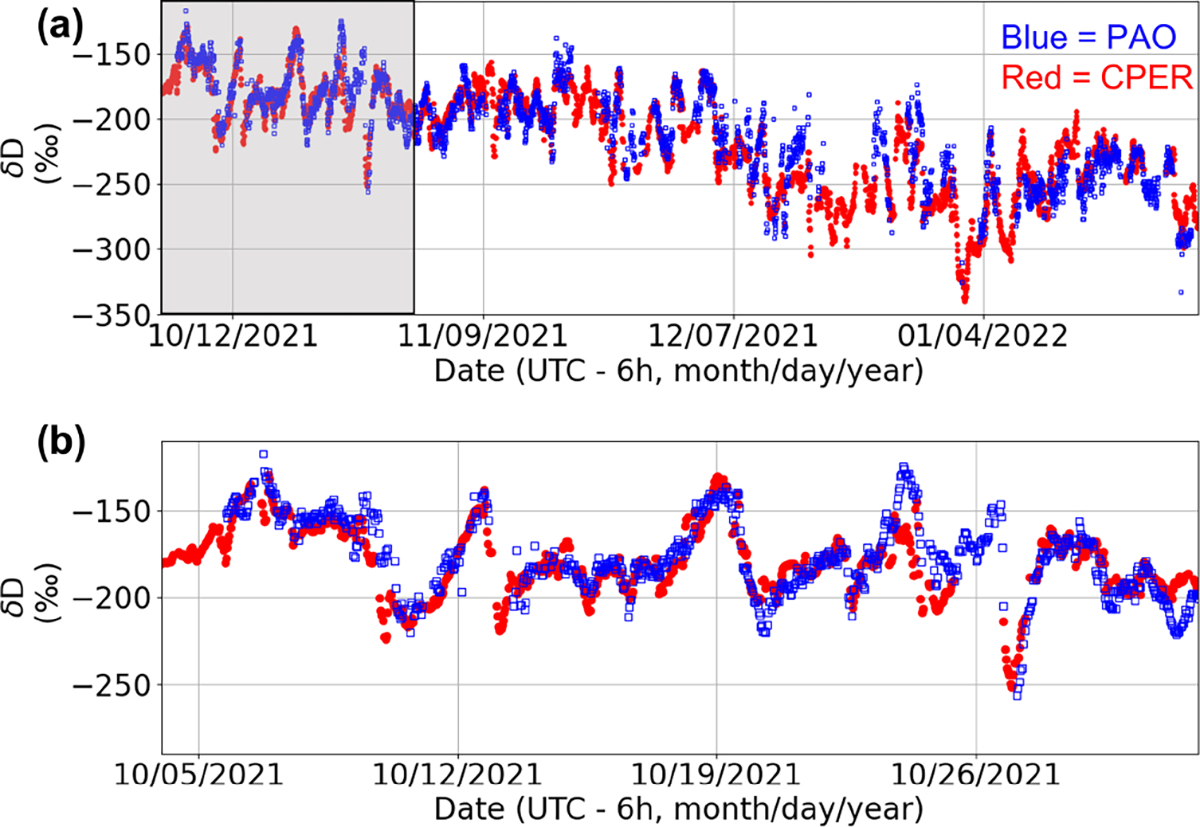
(**a**) Time series of δD over the campaign. Blue squares: open-path DCS at PAO. Red dots: NEON point sensor at CPER. (**b**) Zoomed-in time series of δD for October 2021 (section highlighted in grey in panel a).

Data were acquired from 5 October 2021 to 27 January 2022; time series data are plotted in Fig. 3. The quality of the data is characterized by a DCS figure of merit (FOM), equal to the signal-to-noise ratio at 1 s averaging multiplied by the number of spectral elements (Coddington et al., 2016). Over the 3-month data acquisition, the highest FOM values achieved were in the 1.0 × 10^6^-to-1.5 × 10^6^ range, comparable to recent high-quality laboratory DCS results (Ycas et al., 2018). We only analyze data above a defined FOM threshold of 2.0 × 10^5^, which effectively removes extremely noisy data points with poor return power. For this threshold, our uptime over the multi-month measurement period was 60%. The main cause of dropouts was telescope misalignment and not loss of DCS stabilization or degradation of MIR light generation. The daily average FOM decreased by about 50% over the course of the 3-month-long measurement campaign, mostly due to contamination buildup on the retroreflector.

### 3.2 Temperature correction

**Figure 4.**
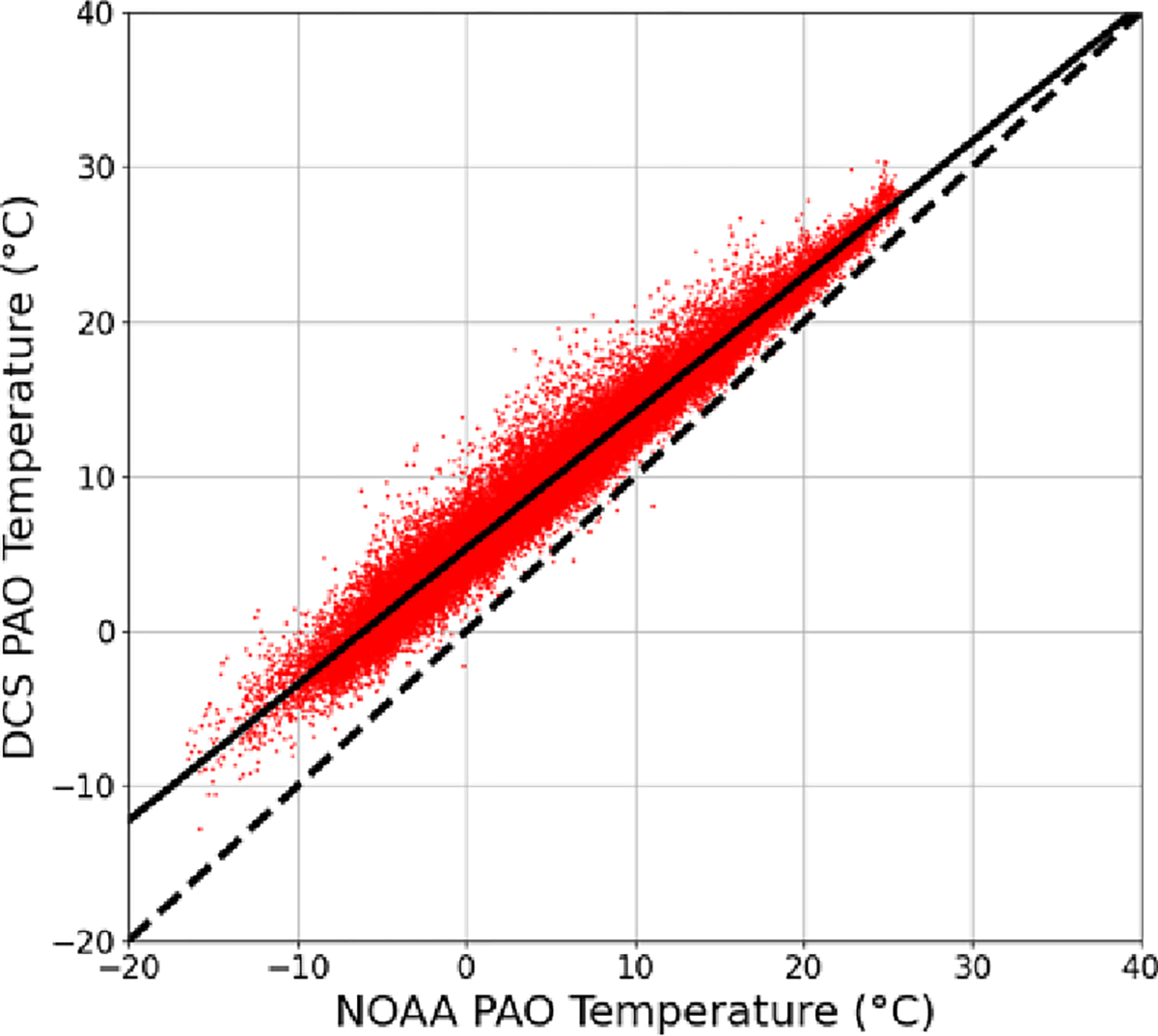
Accuracy of DCS temperature retrieval. Red dots indicate the DCS temperature at PAO versus NOAA temperature at PAO for 2 min windows. A linear fit to these data (solid line) returns a slope of 0.88°C°C^−1^ and an intercept of 5.33°C $\left(R^{2}=0.96\right)$. The dashed line indicates 1-to-1 correlation.

As discussed above, the DCS fit is used to extract a path-averaged temperature. We chose this method over using a separate temperature measurement, since a point temperature measurement does not accurately represent the temperature along the path due to potential horizontal and vertical temperature gradients (Burns et al., 2012). In particular, the NOAA temperature measurement at PAO is at a different height above ground than the optical path, leading to differences due to vertical gradients that undergo a diurnal cycle. However, because the path-averaged temperature is retrieved from the cepstral fit, biases in the spectral database parameters will bias the temperature retrieval. To correct for this, we assume that the DCS and NOAA measurement should agree on average for daily timescales even though there are oscillations on shorter timescales. Figure 4 shows the correlation between the DCS temperature $\left.T_{DCS}\right)$ and the PAO NOAA sensor $\left(T_{PAO}\right)$. The two sensors demonstrate a strong linear correlation, albeit with a non-unity slope and a small offset: $T_{DCS}=c_{0}T_{PAO}+c_{1}$, where $c_{0}=0.88^{\circ }C^{\circ }C^{-1}$ and ${c_{1}=5.33}^{\circ }C$. This bias is not surprising as the HDO line strengths in this region are only known to ~3% accuracy and the broadening coefficients may have up to 10% errors (Devi et al., 2017) (errors in the $H_{2}O$ spectral parameters would also impact T and δD but are generally known to higher accuracy than the HDO parameters). A bias in the retrieved temperature due to spectral database errors will also result in a potential bias in δD. By refitting the same DCS data over a range of fixed temperatures, we find the bias in the extracted δD value, ΔδD, for a given temperature bias, ΔT, has a linear form with $\Delta \delta D=c_{2}\Delta T+c_{3}$, where $c_{2}=10.4‰{\;}^{\circ }C^{-1}$ and $c_{3}=-1.2‰$. By substituting the linear relationship between DCS temperature and NOAA PAO temperature into this equation, we can globally correct the DCS data for the HITRAN temperature dependence errors. The final correction takes the following form:

(2) $$\delta D_{\text{corrected}}=\delta D_{\text{uncorrected}}\;\;-\left\{c_{2}\left[\left(1-\frac{1}{c_{0}}\right)T_{DCS}+\frac{c_{1}}{c_{0}}\right]+c_{3}\right\}.$$

### 3.3 Time series and precision analysis

**Figure 5.**
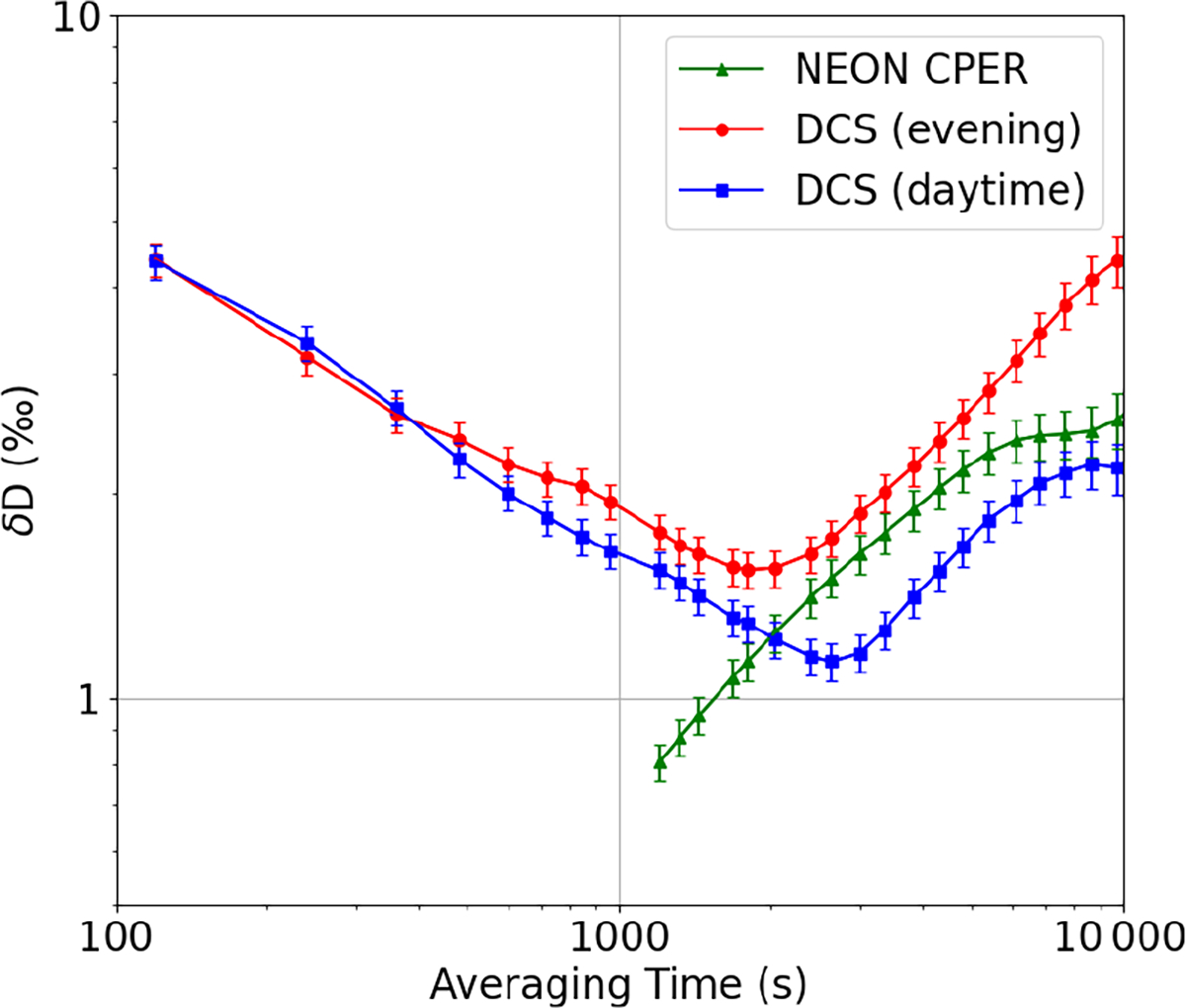
Allan analysis. The overlapping Allan deviation (OADEV) for open-path DCS data from 31 October 2021 when the atmosphere was well mixed (05:20 MDT on 31 October 2021 to 15:30 MDT on 31 October 2021) is shown with blue squares. The OADEV is 4‰ at 120 s and averages down with the square root of time to 1.1‰ at 3000 s. After 3000 s, the OADEV rises steeply, likely due to real atmospheric fluctuations. For comparison, the OADEV for the NEON CPER point sensor is shown for the same period (green triangles). The common upward trend in both indicates variation due to atmospheric fluctuations. Finally, the OADEV for a DCS measurement under more typical mixing conditions (from 15:30 MDT on 31 October 2021 to 01:30 MDT on 1 November 2021) is shown with red dots and exhibits the expected higher instability at longer times due to greater atmospheric fluctuation.

The time series of δD values taken at PAO and CPER is shown in Fig. 3. As will be discussed in depth in Sect. 4.1, the PAO values closely match the CPER values for the entirety of the measurement campaign. We notice significant fluctuation of the δD value over the course of the day as well as slow changes in the average value over the long measurement period. This behavior is consistent with ground-based and satellite-derived δD columns (Schneider et al., 2020; Fiorella et al., 2018; Noone et al., 2013) in the central United States where variations in synoptic weather patterns can bring water vapor from many different climates over the northern Colorado area. To estimate the precision of our open-path DCS δD measurement, we perform an Allan analysis (Werle et al., 1993) of the data taken during the day on 31 October 2021, from 05:15 to 15:30 mountain daylight time (MDT, UTC–6). On this day, the CH_4_ levels were stable and near the minimum observed level (2.02 ± 0.01 μmol mol^−1^) for > 10 h, indicating well-mixed atmospheric conditions. As shown in blue squares in Fig. 5, the Allan deviation reaches a minimum of 1.2‰ in less than 3000 s, after which the true atmospheric variations drive the deviation to higher values. The NEON point sensor at CPER sees a similar level of drift at the hour timescales (green triangles), well above the measurement error of commercial cavity-enhanced systems (Bailey et al., 2015). Furthermore, the Allan deviation of the open-path DCS measurements from a time period with more atmospheric variability (red circles in Fig. 5) shows a minimum at an earlier time, which further indicates that the long-time deviations are driven by atmospheric variability. As we will demonstrate in Sect. 5, single-per-mil precision in less than an hour makes it possible to use open-path DCS to track diurnal and seasonal trends at PAO that match the observed trends from an established point sensor network. This analysis proves that open-path DCS is a viable alternative to extractive point sampling techniques for continuous ecological monitoring of isotopes.

### 3.4 Sources of error in open-path isotopologue sensing with DCS

An evaluation of the accuracy of the DCS δD retrieval by comparison with the CPER NEON site is discussed in Sect. 4.2. In this section, we review the main potential sources of error in the open-path DCS measurement: detection and digitization nonlinearities (Guay et al., 2021; Malarich et al., 2022), concentration retrieval algorithm bias, and spectral model error. To limit the effect of detection and digitization nonlinearities, the received power was limited to < 30 μW and high-linearity amplifiers were used. We have previously confirmed that the detection is linear at these power levels. We also do not observe correlations between retrieved gas concentrations and received power, which indicates linear response.

As with any absorption spectroscopy, the retrieval algorithm can introduce biases depending on the representation of the spectral intensity baseline and treatment of instrumental effects such as etalons. Here, the cepstral technique was used to separate baseline terms from the molecular absorption via filtering in the cepstral domain. The 10 ps cepstral filter was chosen to minimize mean-squared error of the residuals while maximizing the signal-to-noise ratio (SNR). Moving the filter to 5 ps visibly distorts the fit residuals, indicating a poor quality of fit. Moving the cepstral filter to 15 ps causes a 1‰ to 2‰ shift in the retrieved δD and decreases the SNR. Therefore, we can reasonably attribute up to a 2‰ error to the cepstral fit technique.

As discussed in Sect. 3.2, temperature retrieval error has a considerable effect on the retrieved DCS δD value. This correlation is likely driven by the relative temperature dependence of HDO and $H_{2}O$ absorption cross-sections in HITRAN. After performing the linear correction from Sect. 3.2, the residual temperature difference between the DCS measurements and the NOAA PAO sensor is small (±0.5 K, 1σ width). Therefore, the residual systematic error is estimated to be limited at the 5‰ level based on temperature sensitivity of the fitted δD value (i.e., $c_{2}$ from Sect. 3.2). Some of this ±0.5 K temperature difference is likely driven by near-surface temperature gradients (Burns et al., 2012), and therefore the quoted 5‰ error is most likely an overestimate. Although the temperature may also vary slightly across the path, we extract the path-averaged temperature and are mostly insensitive to these gradients (Malarich and Rieker, 2021). In principle, an error in the HITRAN database for the relative pressure dependence of HDO and $H_{2}O$ absorption cross-sections would similarly lead to an error in δD. However, by fitting at different pressures, we find a 1 % error in pressure leads to a negligible 0.1‰ shift in the retrieved isotopologue ratio. Finally, relative error in the HITRAN line strengths for $H_{2}O$ and HDO concentration will correspond directly to an error in δD. As noted earlier, estimated errors for HITRAN are 3 % for HDO and < 1 % for $H_{2}O$ in this region (Devi et al., 2017; Loos et al., 2017), giving an upper limit of 30‰ for the model error in δD. We do compare the absolute $H_{2}O$ retrieval from the DCS system to the co-located CRDS point sensor, finding agreement to within ~1%; however, the CRDS calibration has not been verified, so we cannot quantify this error exactly. Finally, we note that there are slight differences between isotopologue ratios determined using HITRAN and ratios on the VSMOW scale; however, these differences are negligible here (Griffith, 2018).

## 4 Comparison between DCS and NEON sites

### 4.1 Comparison of δD at PAO and CPER

As shown in Fig. 1, the closest and most similar NEON site to PAO is CPER. CPER is about 73 km north of PAO, located near the Colorado–Wyoming border on the northern slope of the South Platte River basin. Both PAO and CPER are located at ~1500 m elevation in high-plains ecosystems (National Land Cover Database grassland/herbaceous class) with cold semi-arid Köppen classification. PAO is surrounded by oil and gas and agricultural development, while CPER is situated far away from most industries and farms.

In general, 20 min average temperatures at PAO and CPER are well correlated with both sites, ranging from −20 to 28 °C during the measurement period. Absolute concentrations of $H_{2}O$ at PAO and CPER are also well correlated, falling within a range from 900 to 12500 μmol mol^−1^ during the measurement period. Because PAO and CPER are located on opposite sides of the South Platte River basin, the two sites do experience differences in microscale and mesoscale meteorology, which leads to a difference in average surface wind patterns (Johnson and Toth, 1982). At the synoptic scale (~300–3000 km scale), the sites are close enough to experience atmospheric flow from mostly similar sources, which was verified using atmospheric trajectory simulation in HYSPLIT (Stein et al., 2015). Thus, we expect that the δD values at PAO and CPER should mostly agree, albeit with some potential differences due to the different micro- and mesoscale meteorology as well as occasional differences in synoptic flow.

**Figure 6.**
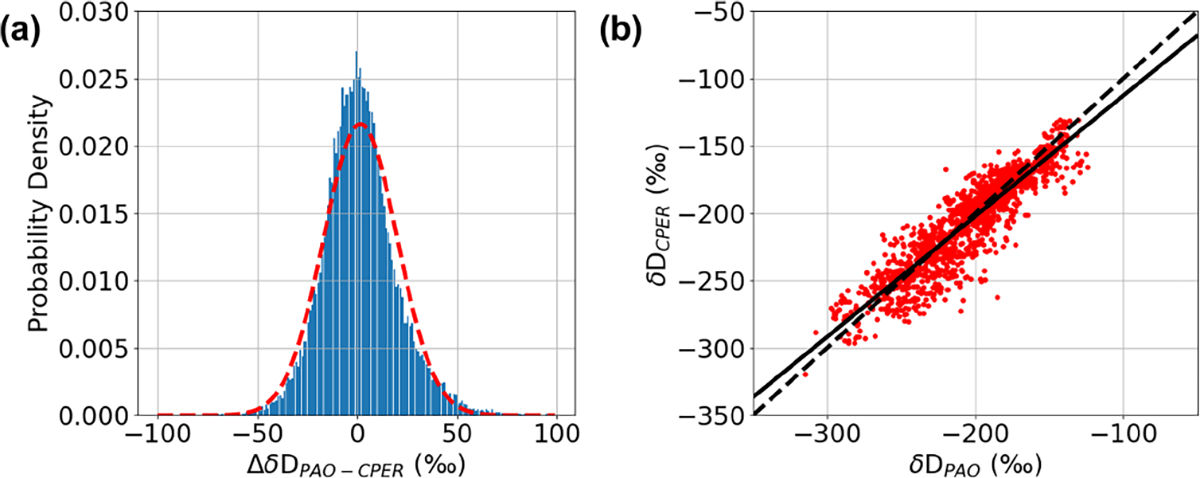
(**a**) Histogram of $\Delta \delta D_{PAO-CPER}=\delta D_{PAO}-\delta D_{CPER}$. Dashed red line: fit to Gaussian model (mean=1.6 ‰, 1σ width=18 ‰). (**b**) PAO δD versus CPER δD. Solid black line: linear fit with slope = 0.90‰‰^−1^, intercept = −23 ‰, and $R^{2}=0.83$. Dashed black line: 1-to-1 correlation line.

The histogram of $\Delta \delta D_{PAO-CPER}=\delta D_{PAO}-\delta D_{CPER}$ over the measurement period is shown in Fig. 6a, and a correlation plot between $\delta D_{\text{PAO}}$ and $\delta D_{\text{CPER}}$ is shown in Fig. 6b. Both analyses validate the accuracy of the DCS data. A Gaussian fit to the histogram data of Fig. 6a yields a mean for $\Delta \delta D_{PAO-CPER}$ of <2‰ and a standard deviation of 18.2 ‰. The slope of the correlation in Fig. 6b is 0.90 with $R^{2}=0.84$. This level of agreement between isotope ratios measured by a point sensor and an active open-path sensor has not been demonstrated previously to our knowledge. The agreement in terms of mean and standard deviation is comparable to or better than observed for vertical column densities measured using solar-looking FTIR and satellite reflectance measurements (Schneider et al., 2020). We note that the $\Delta \delta D_{PAO-CPER}$ distribution does not perfectly follow a normal distribution but is better approximated by a generalized logistic distribution, indicating that the distribution is rather heavy-tailed compared to a normal distribution. This could be from an uncorrected systematic error in one of the measurements or a true difference between isotope values at PAO and CPER.

### 4.2 Comparison of δD at PAO and NIWO

**Figure 7.**
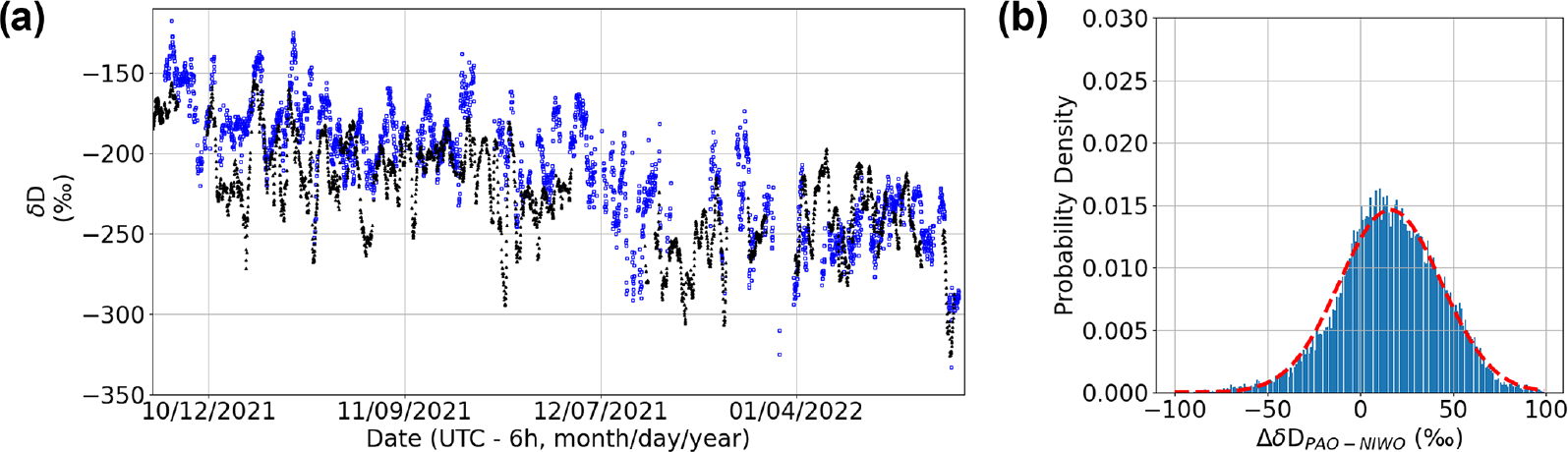
(**a**) Time series of δD at PAO (blue) and NIWO (black). (**b**) $\Delta \delta D_{PAO-NIWO}$ histogram. Dashed red line: fit to Gaussian model (mean = 16 ‰, 1σ width = 27 ‰).

To illustrate the large spatial variability in δD that is possible near the intersection of mountain and plains ecosystems, we also compare the measurements at the PAO site to the NIWO NEON site as shown in Fig. 7. The NIWO site sits at ~3500 m in the Front Range near the Continental Divide about ~20 km west of Boulder and ~75 km west of PAO. The NIWO site has a subarctic Köppen classification and an evergreen forest and grassland/herbaceous class according to the National Land Cover Database. Thus, even though NIWO is located at a similar distance from PAO to CPER, there is a ~2000 m difference in elevation between both PAO and CPER and NIWO, which should lead to higher δD values at PAO relative to NIWO. In addition, NIWO is more strongly influenced by the free troposphere (which has significantly lower δD), leading to time periods with large disagreement between NIWO and PAO. This behavior appears in both the raw times series (Fig. 7a) and the histogram of differences (Fig. 7b). Overall, we observe a ~15‰ offset in $\Delta \delta D_{PAO-NIWO}$. However, the $\Delta \delta D_{PAO-NIWO}$ distribution deviates from a normal distribution with a mode that is closer to a zero offset than the estimated mean. It is likely that the skew and offset of the distribution arise from the varying influence of the free troposphere on NIWO driven by events such as mountain upslope flow (Baumann et al., 1997). This is also observed in the time series where there are time periods with correlated δD values between PAO and NIWO. In the future, DCS can help provide a denser network of measurements at varying altitudes across the Front Range in order to better characterize the atmospheric interactions between the higher-altitude ecosystems and the plains ecosystems below.

## 5 Temporal variability in δD at PAO and CPER

### 5.1 Diurnal and seasonal patterns of δD

**Figure 8.**
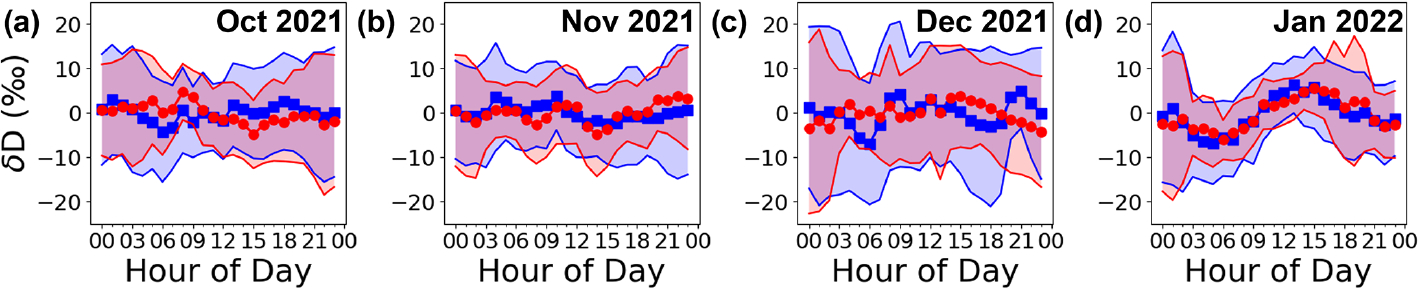
Stacked diurnal cycles for δD at PAO (blue) and CPER (red) with mean value subtracted. Shaded regions indicate standard deviation of values in each hourly bin. (**a**) October 2021. (**b**) November 2021. (**c**) December 2021. (**d**) January 2022.

Diurnal profiles can provide information about local sources of water vapor as well as persistent atmospheric flow patterns such as katabatic winds (Bréant et al., 2019). Here, we show that the open-path DCS measurements have sufficient precision/accuracy to track changes in diurnal patterns of δD. Because significant day-to-day mean variability occurs in δD due to synoptic flow, a stacked or ensemble diurnal profile is generated by first subtracting the daily mean δD and then looking at the diurnal variation in mean-subtracted δD. These profiles are shown in Fig. 8 for each month of the measurement period (October 2021 through January 2022) for both PAO and CPER. As with the histogram, the diurnal profiles generally agree between the two sites, which highlights the similarity between the two locations and shows that the open-path DCS can resolve diurnal patterns. There are some small differences in the diurnal patterns during October and December with PAO showing slightly lower δD values in the morning compared to CPER. This trend could be due to differences in downslope flows at PAO versus CPER (Baumann et al., 1997).

Interestingly, the stacked diurnal profiles show a distinct change between the first 3 months and January 2022. In particular, the January 2022 period shows a clearer diurnal trend that is positively correlated with ambient air temperature. A detailed analysis of diurnal profiles is complicated: in general, the profiles are determined by an interplay between local fluxes (evapotranspiration, sublimation, etc.), atmospheric flow patterns such as mountain flow, and boundary layer dynamics leading to mixing/entrainment with the free troposphere (Welp et al., 2008; Wen et al., 2010; Noone et al., 2013; Su et al., 2022; Steen-Larsen et al., 2013; Bréant et al., 2019). Diurnal patterns with zero or negative temperature correlations like those shown from October 2021 through December 2021 have been observed in both rural and urban environments (Welp et al., 2008; Wen et al., 2010) and were previously hypothesized to be the result of vertical mixing in the planetary boundary layer (PBL) coupled with entrainment from the free troposphere. Diurnal profiles with a positive temperature correlation are often observed in colder climates (Steen-Larsen et al., 2013; Bréant et al., 2019). We do not have enough auxiliary measurements to conclusively identify the cause of this change, but we will note that the period up to the end of December 2021 was extremely dry and somewhat windy and was followed by a snowy and colder January 2022, which suggests that the changes in diurnal profiles might be caused by some combination of meteorological changes related to the colder and snowier weather or larger local water vapor fluxes due to snow cover.

**Figure 9.**
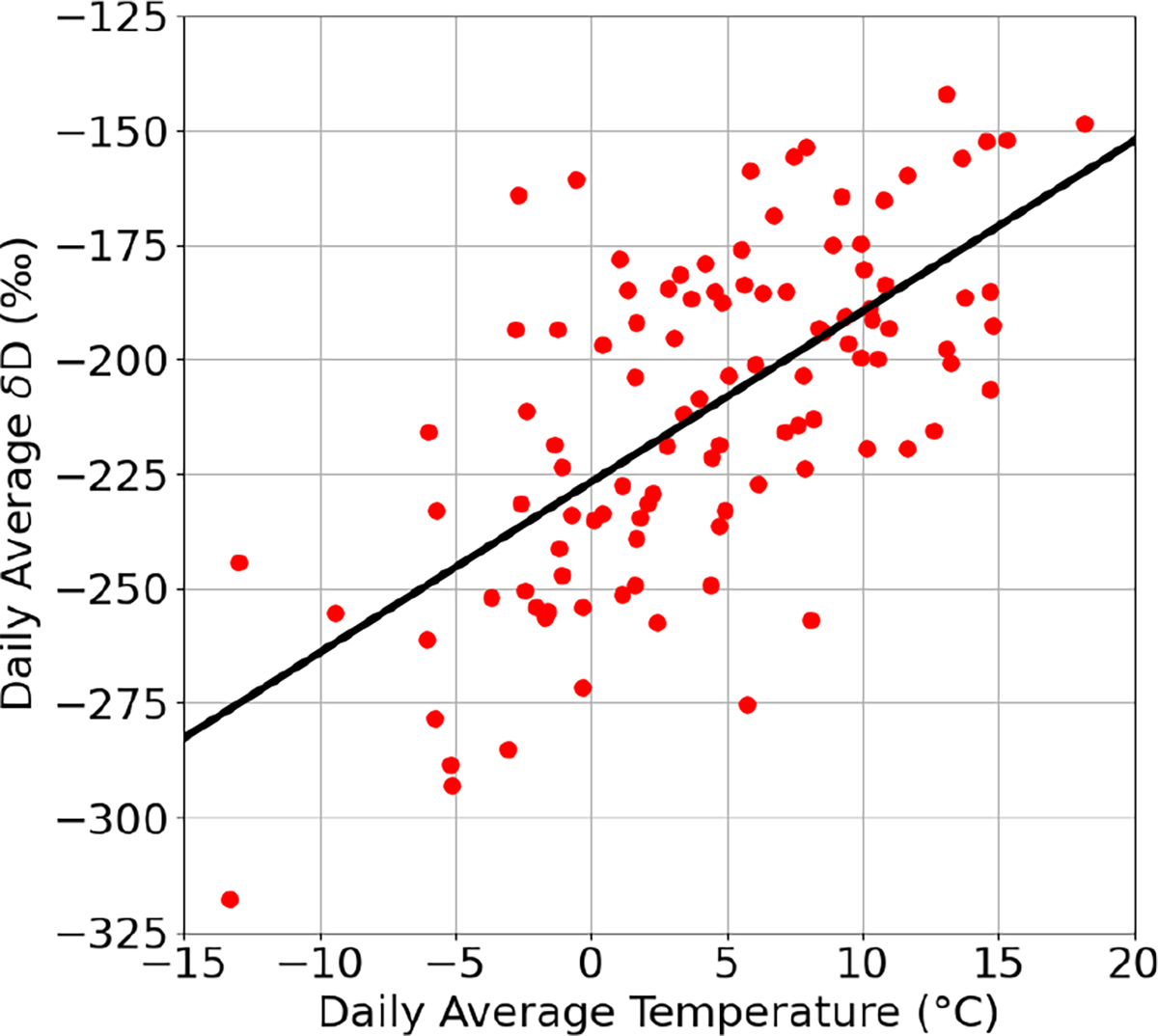
Correlation between daily average temperature and δD at PAO site. Slope = 3.7 ± 0.2 ‰°C^−1^.

The accuracy of the DCS measurements also allows us to track seasonal changes in δD. Unlike the hourly averaged δD, the daily averaged δD trends strongly with daily averaged temperature (see Fig. 9). This correlation holds over the whole measurement period and yields a slope of 3.7 ± 0.2‰°C^−1^, which is consistent with global observations of precipitation in the Global Network of Isotopes in Precipitation (GNIP; Gat et al., 2000). A rolling ordinary-least-squares regression was also performed on the unaveraged δD and temperature data from PAO to confirm the dependence of δD on temperature. This analysis shows that on average there is a 4.1 ± 0.9‰°C^−1^ dependence of δD on ambient air temperature at PAO. The average δD value at 0°C was −227 ± 10 ‰, which is much lower than expected based on temperature effects alone for coastal areas but could possibly be explained by a combination of the altitude and continental effects (Gat et al., 2000; Schneider et al., 2020). Our multi-month measurement campaign has allowed us to view the transition from fall-type diurnal cycles in the Front Range Urban Corridor to winter-type diurnal cycles, with the cycles at PAO and CPER agreeing well both qualitatively and quantitatively without any recalibration necessary for the DCS measurements. The close agreement between the sites suggests boundary layer dynamics and temperature effects are the dominant drivers of δD variation in northern Colorado plains ecosystems.

### 5.2 Temporal differences between PAO and CPER

**Figure 10.**
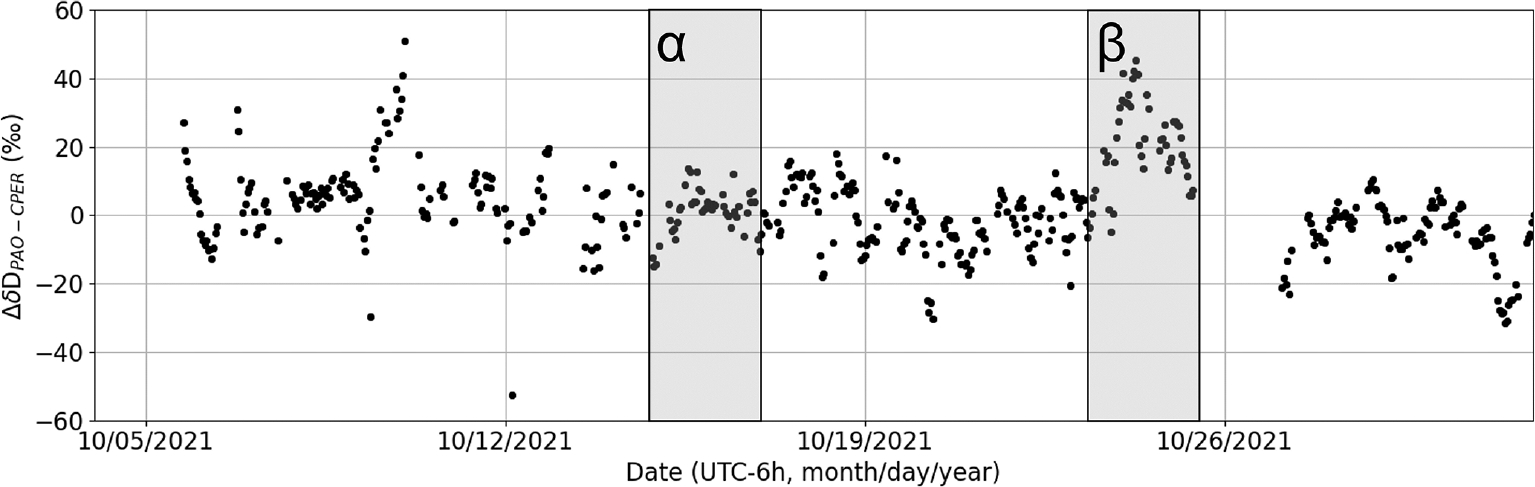
$\Delta \delta D_{PAO-CPER}$ versus time. Very good agreement is observed during the period denoted α (first grey band), while fairly strong disagreement is seen during the period denoted β (second grey band), reaching more than 20 ‰ for about a day.

The high precision of the DCS measurements also provides an opportunity to look for meteorological differences in δD between PAO and CPER. Figure 10 shows $\Delta \delta D_{PAO-CPER}$ over October 2021 resampled to 3000 s time resolution. This month corresponds to the most continuous high-SNR section of PAO data and thus is the best section of data for probing temporal dynamics. In this time series, we see periods with high agreement (e.g., the period marked α spanning 15–16 October 2021) as well as periods with more marked disagreement (such as the period marked β spanning 23–24 October 2021). This disagreement between PAO and CPER is likely indicative of differences in synoptic flow at the two sites during this period as discussed below.

**Figure 11.**
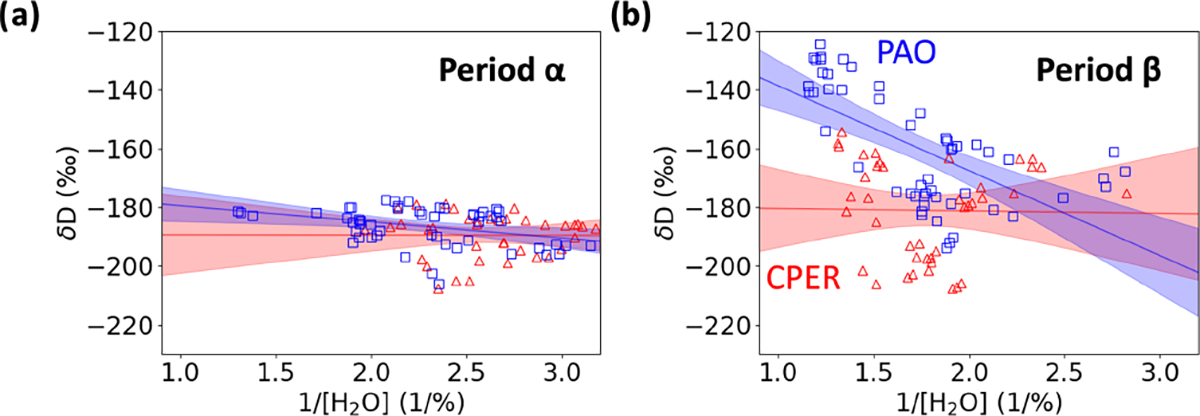
δD versus $1/[H_{2}O]$ over shorter time periods at PAO (blue squares) and CPER (red triangles) suggest intermittent meteorology differences between the sites. (**a**) Data over the time period α of good agreement. The fit to Eq. (3) gives $\delta D_{\text{source}}=-174\pm 9‰$ (95 % uncertainty) for the PAO data and $\delta D_{\text{source}}=-189\pm 21‰$ (95% uncertainty) for the CPER data. (**b**) Data over the time period β of disagreement. The fit to Eq. (3) gives $\delta D_{\text{source}}=-110\pm 19‰$ for the PAO data and $\delta D_{\text{source}}=-180\pm 28‰$ (95 % uncertainty) for the CPER data. Shaded regions indicate the 95 % confidence interval of the linear fit.

Differences in synoptic flow can be probed using a two-source mixing model or Keeling analysis (Noone, 2012; Keeling, 1958). When two air masses with different δD and $H_{2}O$ content mix (e.g., moist ocean air mixing with dry free tropospheric air), δD follows a linear relationship when plotted against $1/\left[H_{2}O\right]$ according to the following form (Noone, 2012):

(3) $$\delta D={[H}_{2}{O]}_{o}\left(\delta D_{0}-\delta D_{\text{source}}\right)\frac{1}{\left[H_{2}O\right]}-\delta D_{\text{source}},$$

where ${\left[H_{2}O\right]}_{\text{source}}$ and ${\delta D}_{\text{source}}$ are the $H_{2}O$ concentration and δD for the source air mass (e.g., moist ocean air) and ${\left[H_{2}O\right]}_{0}$ and $\delta D_{0}$ are the $H_{2}O$ concentration and δD for the dry air mass. We can extract $\delta D_{\text{source}}$ either by plotting δD versus $1/\left[H_{2}O\right]$ and finding the y intercept or by fitting the hyperbolic relation described in Eq. (3). Figure 11 shows comparisons between δD versus $1/\left[H_{2}O\right]$ measured at two times (labeled periods α and β in Fig. 10). During period α, both CPER and PAO show the same mixing behavior; however, during period β, the mixing behavior is significantly different with notably different slopes and intercepts. A limited analysis of atmospheric transport at PAO and CPER suggests that synoptic flow typically comes from the Pacific Northwest, from the southwest, or from a combination of these two. A “latitude effect” has been previously observed where air from higher latitudes tends to be more depleted of heavy isotopologues (Araguás-Araguás et al., 2000; Schneider et al., 2020). During period β, the $\delta D_{\text{source}}$ at PAO was 70‰ higher than at CPER, which suggests that the air at PAO more likely came from the southwest (higher $\delta D_{\text{source}}$), whereas the air at CPER more likely came from the Pacific Northwest or a combination of sources. This provides further evidence that the synoptic-scale atmospheric transport at PAO and CPER can be different.

## 6 Discussion and conclusion

The observed differences in δD between sites separated by 75 km motivate the construction of denser isotopologue measurement networks for benchmarking models of water transport. However, accurate/precise measurement of isotopologues in lower tropospheric water vapor is a challenging task for traditional open-path techniques such as FTIR and continuous-wave (CW) laser spectroscopy. Point sensors can provide accurate measurements but lack the operational flexibility of open-path techniques and require regular recalibration. We have shown that MIR DCS is capable of accurate open-path measurement of $H_{2}O$ and HDO concentrations in real-world settings for extended periods of time. Our DCS δD precision of 1.2 ‰ at 3000 s allows us to characterize shifts in ambient δD levels. After correcting the data for a small linear error in retrieved temperature due to errors in the spectral database, the DCS-based results taken at PAO match those taken at CPER with a mean difference of 1.6 ‰ and a root mean square error of 18 ‰ over a 3-month period. It is likely that some of the differences are true differences driven by meteorology. In the future, laboratory DCS can facilitate improved accuracy for the water absorption lineshape models used to extract isotopologue ratios in the MIR with DCS (Hayden et al., 2019). With the current precision, it should be possible to measure over multiple vertically or horizontally separated paths with one DCS instrument (Coburn et al., 2018; Herman et al., 2021) to understand influences on isotopologue ratios from local evaporative flux (Welp et al., 2008). Such experiments allow for precise extraction of the local isotopologue ratio for the evapotranspirative flux $\left(\delta D_{ET}\right)$ and can be used to better understand the water cycle of natural environments like estuaries or non-natural environments like reservoirs and agricultural operations (Welp et al., 2008; Barrie et al., 2015).

In addition to H_2_^16^O and HDO, open-path DCS simultaneously provides concentration for other atmospheric species and can help answer a variety of questions related to atmospheric transport. Future ultra-broadband DCS systems based on ultra-short-pulsed nonlinear optics (Lesko et al., 2021; Zhou et al., 2020) will enable simultaneous coverage of H_2_^16^O, H_2_^18^O, and HDO for characterization of d-excess (defined as the deviation from global meteoric water line) over open paths (Dansgaard, 1964; Craig, 1961). When combined with information on combustion sources from DCS measurements of ambient CO, CO_2_, and HCHO, the d-excess record may help confirm carbon inventories using open-path DCS systems (Xing et al., 2020). Also, water isotopologue measurements at NIWO and PAO combined with DCS multispecies measurements of trace gases including ammonia, greenhouse gases, and volatile organic compounds could improve our understanding of upslope pollution events that contribute to nitrification and ozone production along the Front Range Urban Corridor and the Rocky Mountain National Park area (Piña et al., 2019; Baumann et al., 1997). We also highlight the important role of ground-based sensors in the calibration of satellite remote sensing measurements of HDO vertical columns (Schneider et al., 2020). In the future, MIR DCS could provide a mobile, reconfigurable platform to supplement the current fixed Total Carbon Column Observing Network (TCCON) and Network for the Detection of Atmospheric Composition Change (NDACC) measurements used for calibration of inversion algorithms used to extract height-dependent water isotopologue ratios (Wunch et al., 2011; Zhou et al., 2023).

## Data Availability

Water vapor isotopologue and meteorological data are available in CSV format through the NIST MIDAS database (https://doi.org/10.18434/mds2-2976, Cossel, 2023).
